# The Arabidopsis Stress Responsive Gene Database

**DOI:** 10.1155/2013/949564

**Published:** 2013-03-17

**Authors:** Subhomoi Borkotoky, Vijayakumar Saravanan, Amit Jaiswal, Bipul Das, Suresh Selvaraj, Ayaluru Murali, P. T. V. Lakshmi

**Affiliations:** ^1^Centre for Bioinformatics, School of Life Science, Pondicherry University, R. V. Nagar, Kalapet, Puducherry 605014, India; ^2^Parasitology Division, Regional Medical Research Centre (RMRC-ICMR), Dibrugarh 786 001, India; ^3^Department of Bioinformatics, SRM University, Tamil Nadu 603203, India

## Abstract

Plants in nature may face a wide range of favorable or unfavorable biotic and abiotic factors during their life cycle. Any of these factors may cause stress in plants; therefore, they have to be more adaptable to stressful environments and must acquire greater response to different stresses. The objective of this study is to retrieve and arrange data from the literature in a standardized electronic format for the development of information resources on potential stress responsive genes in *Arabidopsis thaliana*. This provides a powerful mean for manipulation, comparison, search, and retrieval of records describing the nature of various stress responsive genes in *Arabidopsis thaliana*. The database is based exclusively on published stress tolerance genes associated with plants.

## 1. Introduction

Stress response is the general term for defining the interaction between plants and the extreme environmental conditions. The study of mechanisms of adaptation to stressful and extreme environments provides the basis for addressing environmental, toxicological, and physiological problems [1]. Changes in the expression of individual genes and proteins induced by stress have been monitored under different conditions. As of the year 2000, the sequence of the *Arabidopsis thaliana* genome is nearly completed, and soon a catalog of plant gene expression exceeding a million transcripts will be available [2]. Here, we have listed the stress responsive genes for * Arabidopsis thaliana* (thale cress), a member of the mustard family, that has become a widely used model for the study of plant biology because of its small size, short generation time, facile genetics, and ease of transformation [3].

There are few databases that have been designed for stress responsive genes in plants. Plant Stress Gene Database [4] include 259 stress-related genes of 11 species along with all the available information about the individual genes. While it contains only 33 genes from *Arabidopsis thaliana, *our database contains 637 gene entries related to stress response in *Arabidopsis thaliana*. Another example—STIFDB—Arabidopsis Stress Responsive Transcription Factor Database [5], is a comprehensive collection of abiotic stress responsive genes in *Arabidopsis thaliana*, with options to identify probable transcription factor binding sites in their promoters, which is limited to only abiotic stress. Apart from these, we have The Arabidopsis Information Resource (TAIR) [6, 7], genetic and molecular biology data for the model higher plant *Arabidopsis thaliana*, which is more widespread to different aspects apart from the stress response, which makes it difficult to look for only stress related genes.

We have listed around 44 types of different stress factors related to *Arabidopsis thaliana, *and the database contains 636 gene entries related to stress response with their related information like gene ID, nucleotide and protein sequences, cross-response, and so forth. The database is based exclusively on published stress responsive genes associated with plants. This database also include BLAST [8] search interface for both nucleotide and proteins. The database is freely available and could be accessed via http://srgdb.bicpu.edu.in/.

## 2. Materials and Methods

### 2.1. Data Collection

An extensive literature search has been carried out to identify the potential *Arabidopsis thaliana* genes involved in stress tolerance and stress response. Those literatures that have reported stress tolerance or stress responsive gene have been manually curated to identify the presence of any cross-response. Genomic and proteomic data for the collected gene have been obtained from the TAIR. Apart from these, the database also contains gene expression data obtained from Genevestigator [9] and Arabidopsis Gene Family Profiler (aGFP) [10].

### 2.2. Databse Architecture

**Figure 1:**
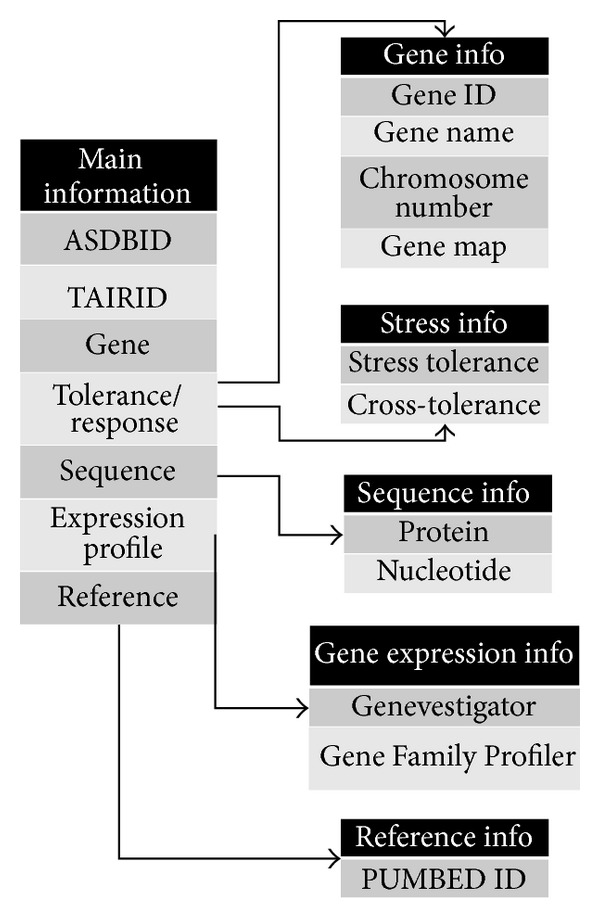
Database structure of ASRGDB.

The Arabidopsis Stress Responsive Gene Database (ASRGDB) has been constructed and configured using typical LAMP (Linux, Apache, MySQL, and PHP) server. Data was stored as a MySQL table using MySQL 5.5, and the MySQL structure model for the ASRGDB is shown in Figure 1.

### 2.3. Database Access

The database can be queried in a various way, unlike the only browsing option in Plant Stress Gene Database. Various keywords, complete or partial, could be searched against the various fields in the database. For easy search, we have included a unique identification number ASDBID to the database. The searchable fields include ASDBID, Response, Cross-response, Gene ID, and Gene name. The results for the search query have been displayed in a user-friendly table view for easy access. A legend is also given in the search tab to guide the users and to directly access the particular stress type with a single click.

### 2.4. Blast Search

The database also has the provision of performing a BLAST search against the ATSRGDB. Both protein and nucleotide sequences can be queried separately. BLAST 2.2.25+ version is used with BLSOUM62 matrix, Gap penalties of 11 and extension 1. The window size for the multiple hit is set to 40 (default). The BLAST hit result is directly linked to the database, and, hence, the hit record can be directly accessed from the BLAST result page.

### 2.5. Database Update

The ATSRGDB also provides interface to update the database. The user can add the new data into the database but not allowed to modify or delete the existing data. The new entry requires the following mandatory field TAIRID, Chromosome number, Response, and Reference. All the entries will be subjected to extensive manual validation. The user has to register to make an entry into database.

Database design and interface has been developed using PHP and MySQL. BLAST searches were carried out using PERL scripts.

## 3. Results and Discussion

The ASRGDB includes a total of 636 records with 44 different types of stress responses. The distribution of different response types in the database is tabulated in Table 1. Salt response and oxidative responsive genes were found to be abundant (139 and 132, resp.), when compared to the other stress types in the database. Out of 636 stress responsive genes, 238 were found to have cross-response with other stress types. Among all, only 9 genes were known not to have coding proteins. The database provides search facility in five different fileds of the database. The database also provides the user to perform a BLAST search against the sequences in the database (both protein and nucleotide).

With this different facility, the Arabidopsis stress responsive gene database stands as a centralized source of information for scientific investigators who are interested in plant stress physiology, mainly *Arabidopsis thaliana*. This database provides a powerful mean for manipulation, comparison, search, and retrieval of records describing the nature of stress responsive genes in *Arabidopsis thaliana*. The database will be updated and curated every six months including individual submissions made by the user. As of future, the addition of more stress-related genes with related information and regular updates with the improvements of several sequencing and analysis techniques will make this database more useful to the research community. 

## Figures and Tables

**Table 1:** Distribution of different stress types in ARGDB.

Response type	Total genes
Salt	139
Oxidative	132
Stress	75
Biotic	10
Drought	31
Osmotic	30
Heat	28
Abiotic	26
Dehydration	19
Cadmium	15
Cold	15
Endoplasmic reticulum	13
Light	11
Abscisic acid	11
Aluminium	10
Water	8
Chitin	3
Salinity response	2
Freezing	2
Genotoxic	2
Photooxidative	2
F box	2
Pathogen	2
Cation	1
Sugar	1
Kinase	1
Phosphate	1
UV	1
Temperature	1
Dessication	1
Ethylene	1
Touch	1
Potassium	1
DNA damage	1
Karrikin	1
Cellular	1
Chilling	1
Hydrogen peroxide	1
Reactive oxygen species	1
Wound	1
Metal	1
Magnesium	1
Sodium	1
Malondialdehyde	1
